# Population analysis of mortality risk: Predictive models from passive monitors using motion sensors for 100,000 UK Biobank participants

**DOI:** 10.1371/journal.pdig.0000045

**Published:** 2022-10-20

**Authors:** Haowen Zhou, Ruoqing Zhu, Anita Ung, Bruce Schatz

**Affiliations:** 1 Department of Statistics, University of Illinois at Urbana-Champaign, Champaign, Illinois, United States of America; 2 Carl R. Woese Institute for Genomic Biology, University of Illinois at Urbana-Champaign, Urbana, Illinois, United States of America; 3 Department of Medicine, College of Medicine, University of Illinois at Urbana-Champaign, Urbana, Illinois, United States of America; 4 Department of Medical Information Science, College of Medicine, University of Illinois at Urbana-Champaign, Urbana, Illinois, United States of America; 5 University Library, University of Illinois at Urbana-Champaign, Urbana, Illinois, United States of America; Tsinghua University, CHINA

## Abstract

Many studies have utilized physical activity for predicting mortality risk, using measures such as participant walk tests and self-reported walking pace. The rise of passive monitors to measure participant activity without requiring specific actions opens the possibility for population level analysis. We have developed novel technology for this predictive health monitoring, using limited sensor inputs. In previous studies, we validated these models in clinical experiments with carried smartphones, using only their embedded accelerometers as motion sensors. Using smartphones as passive monitors for population measurement is critically important for health equity, since they are already ubiquitous in high-income countries and increasingly common in low-income countries. Our current study simulates smartphone data by extracting walking window inputs from wrist worn sensors. To analyze a population at national scale, we studied 100,000 participants in the UK Biobank who wore activity monitors with motion sensors for 1 week. This national cohort is demographically representative of the UK population, and this dataset represents the largest such available sensor record. We characterized participant motion during normal activities, including daily living equivalent of timed walk tests. We then compute walking intensity from sensor data, as input to survival analysis. Simulating passive smartphone monitoring, we validated predictive models using only sensors and demographics. This resulted in C-index of 0.76 for 1-year risk decreasing to 0.73 for 5-year. A minimum set of sensor features achieves C-index of 0.72 for 5-year risk, which is similar accuracy to other studies using methods not achievable with smartphone sensors. The smallest minimum model uses average acceleration, which has predictive value independent of demographics of age and sex, similar to physical measures of gait speed. Our results show passive measures with motion sensors can achieve similar accuracy to active measures of gait speed and walk pace, which utilize physical walk tests and self-reported questionnaires.

## Author summary

Healthcare infrastructure implementation could benefit tremendously from national scale screening with passive monitors. Large scale population data could delineate health risks without intruding into daily living. Digital health offers potential solutions if sensor devices of adequate accuracy for predictive models could be widely deployed. The only such current devices are cheap phones, smartphone devices with embedded accelerometers. This limits measures to motion sensor data collected when the phones are carried during normal activities. So measuring walking intensity is possible, but the total activity measure that is possible with 24-hour wearable devices is not. Our study simulates the use of smartphone sensors to predict mortality risk in the largest national cohort with sensor records, the demographically representative UK Biobank. Mortality is the most definitive outcome, with accurate death records for five years available for the 100,000 participants who wore sensor devices. We analyzed this dataset to extract walking sessions during daily living, then used characteristic motions of these walking sessions to predict mortality risk. The accuracy achieved was similar to activity monitors measuring total activity and even similar to physical measures such as gait speed during observed walks. Our scalable methods offer a feasible pathway towards national screening for health risk.

## Introduction

The association of physical activity with mortality risk is well established. National cohort studies based on self-report have shown intensity to be correlated with survival, as persons who engage in more moderate-to-vigorous activity and less sedentary activity have lower mortality rates [1]. These studies focus upon amount of activity at given intensity level. These findings have been confirmed in large meta-analyses using objective physical activity, in which body worn sensors record total activity and statistical models predict mortality risk using accelerometers [2]. Cohort meta-analyses also show sensor features improve model performance beyond traditional risk factors [3], e.g. smoking and alcohol, independent of demographics, e.g. age and sex.

In addition to the quantity of intensity, there are also implications for the quality of intensity. Physical measurements focus upon walking as a moderate activity, intermediate between vigorous and sedentary activity. Large cohort studies show gait speed is correlated with mortality risk [4], with timed walking over short distances such as 6 seconds for 4 meters. National cohort studies based on self-report reveal walking pace as a unique characteristic beyond traditional demographic risk factors which mediate cardiovascular mortality risk [5]. The 6 minute walk test [6]—where persons walk steadily in hospital corridor, a standard evaluation for cardiopulmonary disease—has been shown in large meta-analysis studies to be a strong independent predictor of mortality from heart failure [7].

Our current study focuses on the largest available national cohort, the UK Biobank [8], where 103,683 participants wore wrist devices with accelerometer sensors for 1 week [9]. In keeping with our previous accelerometer based analysis of the physical activity level of a national cohort [10], the US Women’s Health Initiative, we use raw sensor data during labelled walking sessions to identify characteristic motions for predictive models. This is the first population analysis of walking intensity with mobile sensors, accepting only input types which can be accurately gathered via personal smartphones.

There are four primary methods for measuring physical activity, which all achieve roughly the same accuracy for predictive models of mortality risk. Two methods require active individual participation, such as answering a questionnaire concerning health status (self-report) or walking a fixed distance under observation (gait-speed). These have proven feasible within limited cohort studies, but are problematic to scale for population level assessment, due to logistic difficulty of getting large numbers of people to perform the required tasks on a routine basis. Two measures are passive, collecting data through devices such as activity monitors worn on the body: total amount of physical activity performed during the day and intensity of physical activity such as walking pace over a limited period. These sensor-based methods have the major advantage that they can measure physical activity during daily living, without requiring persons to change their normal activity other than wearing the devices.

However, such digital health approaches have had limited success due to health equity issues relating to access to wearable sensors. Results based on wearable activity monitors come largely from recruited cohorts, such as the nationally representative sample we analyze in this study, rather than from actual community populations. For population measurement in health systems to be routinely available, the measurement devices must be already widely deployed and familiar to the public [11], mandating the choice of mobile phones at present. In the United States for example, the Pew Research Center estimates 97% of the population own cell phones, with 83% possessing smart phones containing motion sensors [12], while only 21% of the population wear sensors such as smart watches or fitness devices [13]. Thus scalable methods for predictive models using mobile phones would have great impact if data analytic limitations can be overcome. Mobile phones are often carried while walking, so they could easily capture walking sessions. Conversely, they are rarely carried all day, so would not be effective at collecting the total amount of physical activity achieved during a day, unlike wearable devices.

The smartphone penetration rate in the United Kingdom, where our dataset was gathered, has increased every year over the past decade, reaching an overall ownership of 92% in 2021. For older adults in the statistical survey [14], less than half of all respondents over the age of 55 owned such a device in 2016, but this total rose to 83% in 2021. Soon adequate devices will be everywhere, with even the cheapest flip phones incorporating motion sensors. Furthermore, inexpensive smartphones are already widespread worldwide, even in the poorest countries [15]. The global smartphone penetration rate is estimated to have reached over 78% in 2020. This is based on 6.4 billion smartphone subscriptions in a global population of 7.8 billion. The global smartphone penetration rate in the general population has great regional variation. In North America and Europe, the smartphone adoption rates are roughly 82% and 78% respectively, whereas in Sub-Saharan Africa, the same rate currently stands at 48 percent. While there is a 30% difference in adoption rates between the highest and lowest ranked regions, note that even in low-income regions half the population already has smart phones with motion sensors.

Cheap phones could have major impact in addressing health equity if proper models can be developed to utilize the limitations of the data provided by their sensors when they are carried. Our study uses the sensor dataset from the largest current national cohort, the UK Biobank, the largest sensor dataset currently available. Although this data was gathered from activity monitors, our sensor models use only the inputs that would be feasible to gather using inexpensive, currently available, phones. This is possible because of our extensive clinical experiments with cheap phones, developing highly accurate predictive models for health status for cardiopulmonary patients [16]. In addition, the 100K participants included in this paper are demographically similar to the overall 500K UK Biobank participants, who match the characteristics of the national population [17], thus providing significant generality to the model results.

## Results

We show short bursts of steady walking suffice for predictive models of mortality risk, evaluated using raw sensor data for 100,000 participants in UK Biobank. Our Results evaluate the model accuracy for mortality risk using walking intensity, defined as 12 walking windows of 30 seconds each during a consecutive session, representing daily living versions of walk tests. Our accuracy is comparable to previous models using daily profiles of total activity. Our methods are logistically easier, with 6 minutes per day (12 windows) rather than 600 minutes (10 hours) per day of sensor records. Although the analyzed dataset uses wrist sensors, our previous work showed cheap smart phones have good enough accelerometer sensors to be accurately utilized for similar analysis of walking sessions [18]. Our clinical studies have shown predictive models using only walking intensity can accurately compute pulmonary function for cardiopulmonary patients [16]. Thus our analysis with wearable sensors for predicting mortality is directly applicable for clinical practice with personal smartphones, already ubiquitous in the UK and the US populations, and widespread in global populations.

### Max (maximum) models

To model mortality, we consider maximum follow-up time of 1/2/3/4/5 years. This means when the maximum follow-up time is 1 year, any event after 1 year after sensor records is ignored. So we can evaluate model accuracy in early risk years, as well as standard 5-year mortality. The highly accurate UK Death Registry is used to determine which participants had died by that time.

As detailed in the Methods section, we choose 20 traditional predictors, from self reports and laboratory tests. These 20 questions are listed in Table 1 as the Categorical Features. The full encoding from UK Biobank data fields is given in S1 Table.

**Table 1:** Categorical Features from Question Answers in UK Biobank data fields.

Demographics	
Age/Sex/Race	Age = DateSensorWear-YearOfBirth Sex = Male/Female, Race = Other/White
**Diagnosis**	
Cardiovascular Disease	combine 8 fields, eliminate duplicate participants.
Myocardial Infarction, Angina Pectoris	Congestive Heart Failure, Ischemic Heart Disease
Stroke Cerebral Infarction, StrokeOther	Stenosis Precerebral Arteries, Cerebral Arteries
Pulmonary Disease	Chronic Obstructive Pulmonary Disease (COPD)
Cancer	Date Diagnosed with Malignant Behavior
Diabetes	combine 3 fields, Type 1 + Type 2 + Other
**Medical Care**	
Operation	Major Operation for Male + Female
Hospital Admissions	Emergency Admission, Elective Admission
Falls	Falls in Last Year, High risk / Low risk
**Screening**	
Hypertension	Essential Hypertension from Physician Panel
Cholesterol	Total Cholesterol, Normal< = 5.9
Obesity	Body Mass Index (BMI), Normal<30
Medications	Number of meds, Good<5
**Habit**	
Alcohol (Intake Frequency)	Daily, 3-4/Week, 1-2/Week, 1-3/Month, Special, Never
Smoking (Current Tobacco)	Yes (Bad), Occasional, No (Good)
Stress (Illness/Injury/Grief)	Serious You (Bad), Serious Other, None (Good)
**Lifestyle**	
Health (Overall General Health)	Excellent/Good/Fair/Poor
Education (Schooling/Training)	None = 1; Any value (college, professional)
Income (Average Household)	0-18K, 18-31K, 31-52K, 52-100K, >100K Pounds/Year

ADC Advanced Disease Condition is 7 features (4 Diagnosis + 3 Medical Care)

MRF Modifiable Risk Factors is 10 features (4 Screening + 3 Habit + 3 Lifestyle)

We also choose 76 derived predictors from motion (accelerometer) sensors. These 76 sensor features are listed in Table 2 as the Continuous Features. The full encoding from UK Biobank software [19] is given in S2 Table.

**Table 2:** Continuous Features from Sensor Records via Participant Accelerometers.

ENMOtrunc	Euclidean Norm Minus One truncated (make zero if negative)
ENMOabs	Euclidean Norm Minus One absolute (make positive if negative)
Mean	mean of vector magnitude (+x/y/z axis)
Sd	standard deviation of vector magnitude (+x/y/z axis)
RMS	Root Mean Square (quadratic mean, average power) (+x/y/z axis)
TAC	Total Activity Count (total motion amount) (+x/y/z axis)
MCR	Mean Crossing Rate (across signal average) (+x/y/z axis)
MMCR	Average of Min-Max Crossing Rate (+x/y/z axis)
MAD / MPD	Mean Amplitude Deviation / Mean Power Deviation
Min / Max	Minimum / Maximum vector magnitude (+x/y/z axis) 25thp/Median/75thp
Range	total range of x/y/z axis
kurt/skew	kurtosis / skewness (shape of distribution)
CoV	CoVariance in xy/xz/yz axis
corr	Correlation in xy/xz/yz axis
autocorr	autocorrelation of acceleration (time series) (+x/y/z axis)
coefvariation	coefficient of variation (relative dispersion) (+x/y/z axis)
pitchg/rollg/yawg	magnitude (gravity) of accelerometer rotation (3D)
sdpitch/sdroll/sdyaw	standard deviation of accelerometer sensor rotation
avgpitch/avgroll/avgyaw	average of accelerometer sensor rotation

76 Variables for Sensor Features: Accelerometer Derived in Time Domain

38 computed using Biobank software on field 90001

38 further derived using Biobank frequency domain

*Biobank* software: ENMOtrunc, Mean/Sd+x/y/z (9)

MAD/MPD, kurt/skew (4)

Min/Max, 25thp/Median/75thp, autocorr/coefvariation (7)

Range x/y/z, Cov yz/xz/xy, corr yz/xz/xy (9)

pitch/roll/yaw g/sd/avg (9)

*Derived* Software: ENMOabs (1)

x/y/z for Min/Max, 25thp/Median/75thp, autocorr/coefvariation (21)

new features computed: RMS/TAC/MCR/MMCR +x/y/z (16)

**Fig 1:**
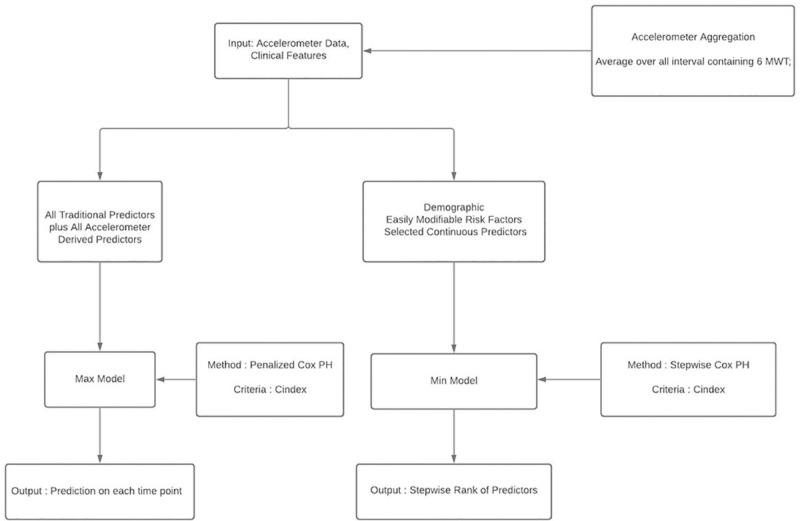
Model flowchart. Which inputs used for which models.

We fit a penalized Cox proportional hazard model with all these features, and denote this the *Max Model*, which evaluates accuracy with maximum functionality. Fig 1 gives the computation flowchart for predictive models.

**Fig 2:**
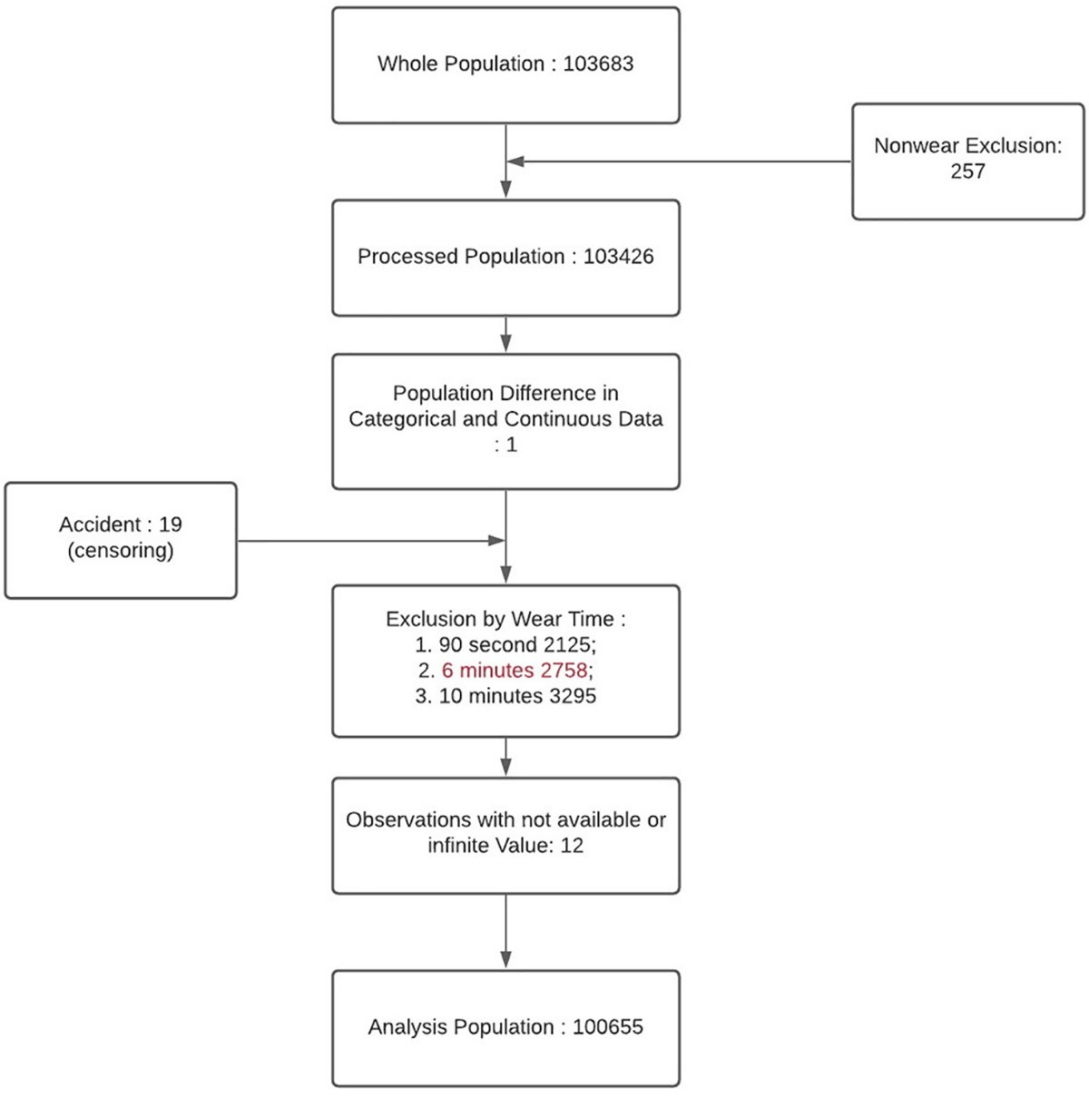
Participant flowchart. Inclusion/Exclusion for Mortality Prediction.

We computed Max Models for different groups of categorical features. All the Models included the 3 demographic features (age/sex/race). The continuous variables are all 76 sensor features, with only steady walking as model input. Fig 2 shows that 100,655 participants met the inclusion criteria of 1 walking session of 6 steady minutes during the 1 week.

**Fig 3:**
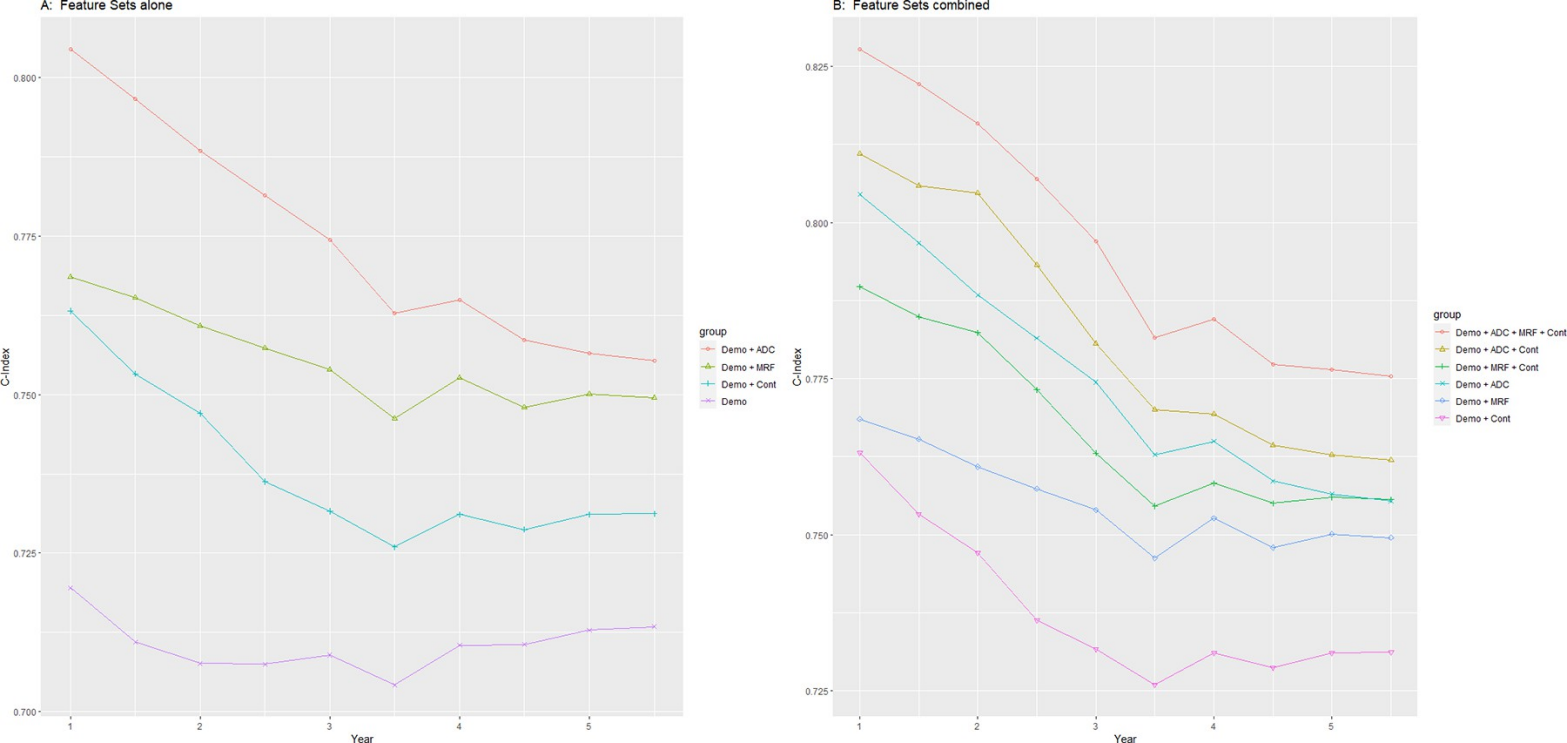
Max model plots with features sets and demographics. Demo is Demographics of age/sex/race. Cont is Continuous (Sensor) Features. MRF is Modifiable Risk Factors (Categorical) Features, ADC is Advanced Disease Condition (Categorical) Features.

The Max Model plots are shown in Fig 3. The plot showing continuous variables alone is given in Fig 3A, where continuous is a distinct improvement on demographics. The C-index is 0.76 at 1-year risk, falling to 0.73 at 5-year risk. The modifiable risk factors are similar at 1-year, where the sensors are more recent, but slightly better at 5-year risk. The advanced disease are significantly better even more at 1-year, but converge to the same as risk factor at 5-year risk.

The continuous variables always improve the accuracy of any set of features, as plots show in Fig 3B. Continuous features with demographics only at the bottom is 0.76 at 1-year and 0.73 at 5-year. Whereas continuous with all categorical features at the top is 0.83 at 1-year and 0.78 at 5-year. In-between, continuous slightly improves the curve for risk factors and the curve for advanced disease. The C-index evaluation numbers of all the Max Models are given in Table 3.

**Table 3:** Max Model with C-Index Results. Feature Sets versus Risk Years.

FeatureSet / RiskYear	1	2	3	4	5
Demographics	0.720	0.708	0.709	0.710	0.713
advanced_disease	0.748	0.739	0.714	0.699	0.682
risk_factor	0.720	0.715	0.702	0.694	0.687
demo + advanced_disease	0.805	0.788	0.774	0.765	0.757
demo + risk_factor	0.769	0.761	0.754	0.753	0.750
risk_factor + advanced_disease	0.794	0.785	0.760	0.747	0.732
demo + risk_factor + advanced_disease	0.822	0.806	0.791	0.780	0.772
Continuous	0.688	0.693	0.684	0.676	0.671
demo + continuous	0.763	0.747	0.732	0.731	0.731
demo + advanced_disease + continuous	0.811	0.805	0.781	0.769	0.763
demo + risk_factor + continuous	0.790	0.782	0.763	0.758	0.756
demo + advanced_disease + risk_factor + continuous	0.828	0.816	0.797	0.785	0.776

Continuous variables have many similarities. Sensor features extracted from signal processing on raw accelerometry compute the same input with similar outcomes on predictive models. Each feature does provide additional accuracy, as shown in S3 Table, which gives marginal performance of each feature by itself. The top features after Age are ENMOtrunc and MPD, along with their variant computations ENMOabs and MAD. These are features measuring the average acceleration of sensor signal via Euclidean Norm or Mean Deviation. ENMO is Euclidean Norm Minus One, after adjusting acceleration for effects of gravity [20].

**Fig 4:**
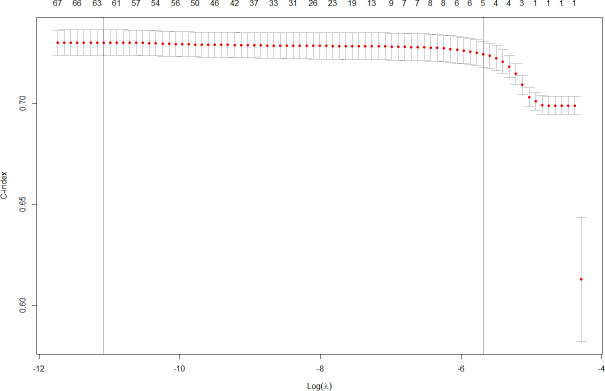
Feature selection for Min Model. Lasso lambda versus Features impact.

Lasso models can be utilized to select fewer features, by focusing on those most predictive [21]. The additional discrimination provided by each feature flattens out quickly, so 5 features provide the same model accuracy as all 76 features, for cross-validated C-index. This is shown in Fig 4, which shows cumulative effort of multiple features flattening after 5 features.

A Lasso model average hierarchy is displayed in S1 Fig, with 16 selected features displayed in red. This model uses the optimal lambda selected from cross-validation. The tree is constructed using the hierarchical clustering algorithm to group sensor features, so that features in similar branches of the tree have similar contributions. At the center of the tree are the acceleration magnitude sensor features–enmoTrunc and enmoAbs, plus MAD and MPD. The red ones are stronger, so enmoTrunc and MPD sensor features, among the discriminating ones, are the best candidates for continuous features. These measure mean and standard deviation of average acceleration, shown to be strongly correlated with intensity of activity [22]. ENMO uses the magnitude of the acceleration and MxD uses the signal of the accelerometer.

### Min (minimum) models

Feature selection implies a parsimonious model might be equally accurate and thus more practical, since requires less input and less compute. Hence, we explore the stepwise model strategy that utilizes small numbers of features. We denote this the *Min Model*, as shown in the flowchart in Fig 1. Such models include demographics and selected continuous features, so include sensor input only with no categorical features. Min Model values are given in Table 4.

**Table 4:** Min Model sensor input only, with cumulative C-index results.

	Name	Cumulative
1	Age	0.699318
2	ENMOtrunc	0.713577
3	Sex	0.727189
4	xSd	0.727784
5	Mean	0.727859
6	ySd	0.727928
7	Sd	0.728136
8	ENMOabs	0.727939
9	Race	0.727893
10	yMean	0.727674

Choose top 10 most discriminating from all

76 sensor features, plus 3 demographics (age/sex/race).

Cumulative C-index for demo+continuous for 5-year risk.

We rank order the top 10 features, after considering all 76 features. The top accelerometer feature is ENMOtrunc, acceleration magnitude correlated with activity intensity, truncated to zero for negative values [22]. With the significant demographic variables Age and Sex, the C-index is 0.727 for Min Model, rounding to 0.73, the same as the Max Model. The remaining top 10 had little extra effect, and may even decrease the cross-validation error. These features include other mean and standard deviation of acceleration.

### Demographic independence

**Fig 5:**
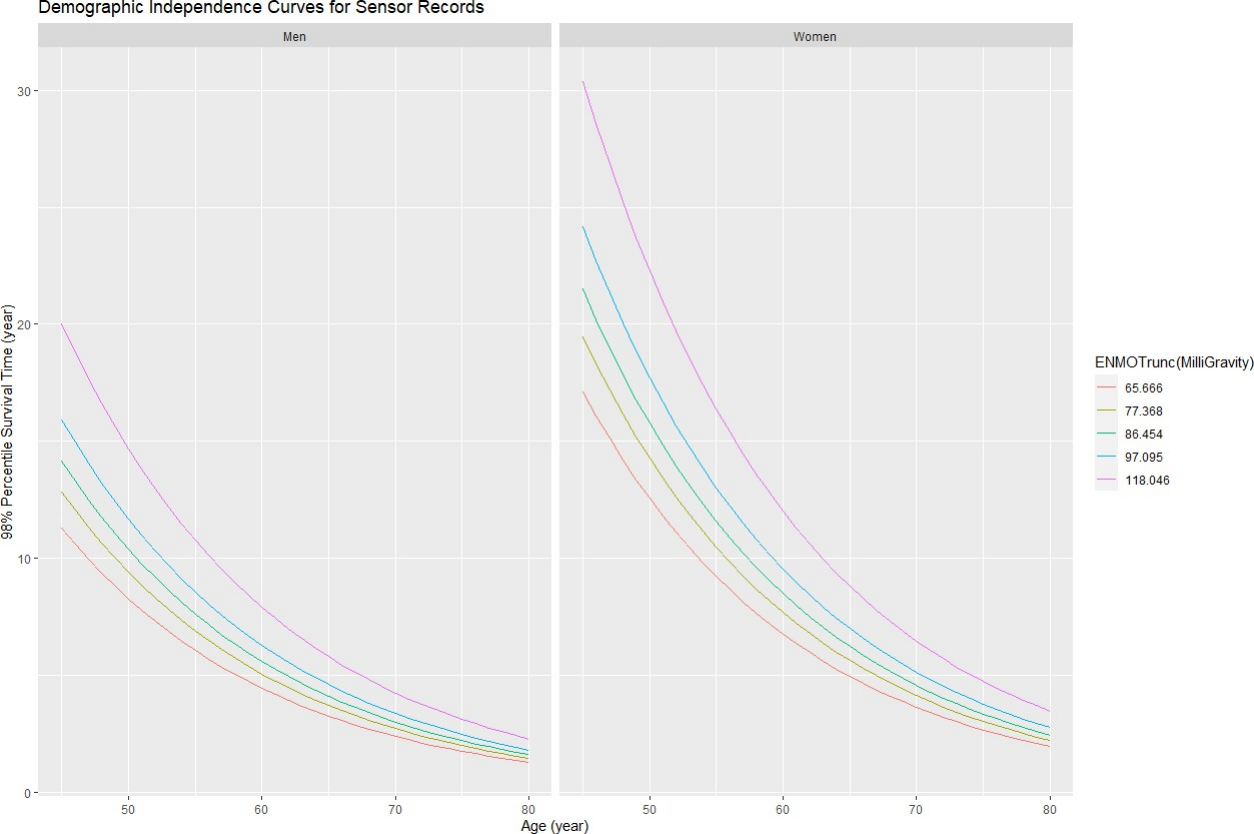
Demographic independence curves for sensor records. Acceleration magnitude (ENMOtrunc) independent predictor against Age and Sex.

For sensor features to provide clinical utility, they must provide orthogonal support, for model accuracy independent of demographics. Following the original gait speed study [4], we generated the curves of 98% percentile survival time as functions of ENMOtrunc against Age and Sex. Death events are about 2% of the cohort. Thus we utilize average acceleration as a surrogate for gait speed. These mortality curves are shown in Fig 5, demonstrating sensor features provide independent value for the practical subset of the Min Model. Higher ENMO values predict longer survival, independent of age and sex. For both men and women, survival declines progressively with age, but each higher level of ENMO sensor feature improves survival rates. The other acceleration magnitude features (MPD/MAD) have similar independence graphs. So the activity intensity predictor ENMO predicts mortality risk—higher magnitude is lower risk.

### Geographic variation

Over the entire cohort of 100K participants, the predictive model gives 0.73 C-index for 5-year mortality. However, it is worth noting that different populations have different accuracies using the same models. To evaluate this, we computed different statistical models (Lasso, Stepwise), then computed the C-index independently for each assessment center in 22 cities across the UK. Accuracy varies widely, as shown in S2 Fig. For example, the Lasso model averages 0.77 but ranges from 0.73 to 0.84, Lasso Continuous averages 0.73 but ranges from 0.66 to 0.77 C-index. With just Continuous, like the Min Model, Glasgow and Edinburgh have 5-year 0.77 C-index. The original mortality study without sensors had its highest reported scores at the Scottish sites as well [23]. Their analysis with categorical features only showed Glasgow and Edinburgh had 0.80 C-index with 13 questions while our categorical only is 0.81 and 0.80 with 17 questions. So similar accuracy with similar set of questions.

### Intensity versus duration

As noted, physical activity is traditionally measured by total duration. Thus individuals with lower mortality have more moderate-to-vigorous activity and less sedentary activity. That is, the duration of activity, the total volume, is considered more important than the intensity itself [24]. Such studies of physical activity commonly utilize wearable sensors since they are specific measures within limited periods [2]. The participants can thus be relied upon to wear the devices all day, so the studies assume 10 hours per day of wear time during normal activities. For effective usage in daily living, the patients must continuously wear a medical quality sensor device. In contrast, our methods assume a single 6MWT per day, so 6 minutes rather than 600 minutes, two orders of magnitude less sensor data. Our methods enable studies with cheap smartphones, since often carried while walking yet having adequate accelerometers for predictive models of pulmonary function [16].

With cardiopulmonary patients, intensity is more important than duration, as shown in large meta-analysis studies [25]. Our model prediction relies upon walking intensity in short bursts being an effective surrogate for activity intensity over whole days, same as the base assumption for walk tests. Intuitively, walking is the unique physical activity, which ranges in intensity from vigorous (fast) to sedentary (slow). Brisk walks are nearly as vigorous as running and shuffling walks are nearly as sedentary as standing.

Another confirmation of walking intensity as an effective surrogate for activity duration is shown by our Lasso model average hierarchy in S1 Fig, with the most discriminating selected sensor features displayed in red. The center of the tree is the acceleration magnitude features such as ENMO and MPD. In a single central branch for selected features are MCR, Mean Crossing Rate, along with MMCR (Maximum and Minimum average Crossing Rate), the closest equivalent with walking intensity to RA (Relative Amplitude) which measures activity duration, as described below. In addition to overall average for MMCR, nearby branches include in red, the specific yMMCR and zMMCR due to walking motions. But the other 4 features in red near the center are the equivalents of RA for short bursts, which we computed for comparative purposes as special additions to Biobank features. These concern the crossing rate, how often the acceleration changes from above the mean to below the mean and vice versa, which we previously utilized for predictive models of fall risk in national cohorts [10].

### Comparing accuracy to concurrent study

The UK Biobank accelerometer dataset has also been analyzed by a concurrent study, performed at the same time by another group separate from our group. This easier study used processed data to analyze the activity duration [26], rather than using raw data to analyze the walking intensity as we did. Their study took the 5 second averages from Biobank field 90004 and further averaged over 1 minute intervals. Our study used raw signals from field 90001, which can detect characteristic motions of walking intensity, since 1 minute contains 6000 data points rather than only 1. Our Min Model achieves the same C-index accuracy of 0.72 as their study for continuous features with demographics, although their analogue of ENMOtrunc called Relative Amplitude (RA) requires 100 times more minutes per day. RA compares the highest 10 hours of activity to the lowest 5 hours, so needs 600 minutes rather than 6 per day. Since they are measuring duration (quantity) of physical activity, they average the sensor records to 1 value per minute of accelerometer data. Since we are measuring intensity (quality) of physical activity, we use the raw data at 100 Hz which is 6000 values per minute. So each day, we measure 100 times less minutes with 10 times more samples. Due to their requirement of at least 3 days with at least 10 hours per day sensor records, they excluded 21K participants while we excluded 3K participants, so we have 7 times more inclusions for measured participants.

In our study, we used Date fields giving physician consensus for disease diagnosis, more detailed from more sources than the participant self-reported answers used in the concurrent study. The Diagnosis features are the most discriminating of all the categorical features, and the Date fields were generated around the same time as the sensor records. This enables a fair comparison between categorical and continuous features, both up to date at the time of sensor records. We took the rest of the categorical features from self-reported features. The self-reported features are from participant registration, which is 6 years before the sensor records (2008 versus 2014, for categorical 2006–2010 and continuous 2013–2015). The concurrent study used only self-reports for categorical features, even for the most discriminating diagnosis features. So the concurrent study has a recency problem with the dataset. Their sensor features are more recent than their categorical features, and hence more accurate for mortality prediction than is actually correct, similar to 5-year versus 10-year mortality risk. Their primary conclusion that sensor features improve prediction performance more than risk factors thus is flawed, a confounded artifact of when data was gathered. As our Fig 3 shows, categorical features actually improve prediction more than continuous features. Our study does not directly compare continuous to categorical features to avoid this artifact.

## Discussion

Measuring physical activity via walking intensity has become a standard practice for certain clinical settings, where gait speed can be quantified with a short walk. Detailed meta-analyses showed that gait speed is a predictor independent of age/sex [4], with a pooled C-index close to 0.72 model accuracy for 5-year mortality risk. Other metrics like Objective Physical Activity (OPA) look at the “quantity” of physical activity, such as total amount of moderate-to-vigorous physical activity, requiring sensor devices to be worn all day. For example, the concurrent study [26] of the same UK Biobank sensor dataset developed a model where the highest predictor of mortality was relative amplitude (RA), the ratio of the most active 10 hours of average acceleration to the least active 5 hours. The C-index studied was 0.72, with RA plus age/sex for 5-year risk, based upon 600 minutes per day of sensor records.

A walk test measures “quality” (intensity) rather than “quantity” (duration). Our previous work showed accelerometer sensors in carried smartphones can digitally model physical distance [27] and oxygen saturation [28] during a Six Minute Walk Test (6MWT). We also showed that the pulmonary models similarly worked with smartphones carried during daily living [16]. The logistical advantage of using 6 minutes of walking intensity is two orders of magnitude less frequent sensor input, using ENMO for quality instead of RA for quantity. Measuring intensity/quality makes it possible to effectively utilize smart phones instead of wearable sensors for predictive models.

Our Min Model with only sensor features holds at the same C-index of 0.72 for 5-year mortality risk. For continuous features only without categorical features, our Max Model with all the sensor features yields 0.73 C-index for 5-year risk. This model has greater accuracy in earlier years yielding 0.76 for 1-year risk. We note model accuracy varies by local sites, as shown in S2 Fig, with 0.77 for 5-year risk at the Scottish sites of Glasgow and Edinburgh, where the original mortality study using self reports also did best [23].

There are significant limitations to our current research. The most obvious is that the UK Biobank dataset was generated by wrist-worn motion-sensors. The sensors themselves are equivalent to those contained in smartphones, but the wearing patterns may not be, so results may differ when large datasets generated by personal smartphones become available. The walking patterns of large populations chosen for health equity may also differ, since low-income lifestyles differ from high-income lifestyles even when the demographics of age and sex are the same, as in the UK Biobank dataset. The methodology of what is considered to be walking sessions might be thus affected, since 6 minutes of steady walking was chosen to mimic walk tests for hospital patients with cardiopulmonary diseases. Our models computed all-cause mortality of patients aged 45–79 for the 5 years past when sensors were recorded. Utilizing walking intensity implies that higher predictive accuracy might be achieved for older patients only, especially those who ultimately die from cardiopulmonary diseases where characteristic motions are more discriminating than with other causes of mortality. We have planned large population trials with only cardiopulmonary patients carrying their personal smartphones, to investigate whether such walk test cohorts produce more accurate predictive models. We hope our research makes clear that large trials employing passive monitors with diverse populations using cheap phones are now technically feasible and socially desirable.

In terms of future directions, we are involved in planning the physical activity study for the US Precision Medicine Initiative (All of Us Research Program), especially the use of phones for health monitoring. This historic longitudinal cohort is planned to have more than 1M participants and is already over 50% enrollment. Participants are being recruited to be representative of the US national population, which is considerably more diverse than that of the UK. For example, the ethnicity “white” covers 94% of all UK Biobank participants, so “race” is weakly correlated with mortality risk in our current analysis. Race/ethnicity is more easily stratified with the US Precision Medicine Initiative population. All consenting participants would be longitudinally measured on their personal smartphones, directly utilizing smartphone sensors, with both a larger sample size and a longer time horizon than our current mortality analysis.

Our previous work showed accelerometer motion sensors in cheap smart phones can capture predictive model input for walking intensity analysis equivalent to expensive medical devices. This is particularly important for health equity purposes, given populations at highest health risk are often the least resourced—so persons most likely to have cheap phones rather than wearable devices would benefit most from easy assessment. Phone apps could record six minutes of consecutive walking during daily living, then compute predictive models for risk stratification via population analysis [11]. To test this strategy, we have planned large trials with minority populations using personal smartphones, within the US Hispanic Community Health Study. Our results from high-income countries may be directly applicable to low-income countries. Major cohort studies using self-reported status have shown cardiovascular health is strongly correlated with physical activity, largely independent of the socioeconomic level of the participants country [29]. Healthy longevity can be facilitated globally for all adults possessing cheap phones, using the minimum model to assess gait status, computed on their phones for the maximum privacy. Implementing effective healthcare infrastructure requires continued research into screening populations with ubiquitous sensors [30].

## Materials and methods

### Ethics statement

This study analyzes datasets provided by UK Biobank, with subjects identified only by participant number. This Biobank is a national resource in the United Kingdom, providing datasets to international researchers who have approved projects. Our project entitled “Predictive Models of Mortality Risk from Passive Monitors measuring Physical Activity” is approved with ID 45178. This enabled us to download datasets with selected portions of their complete database, each dataset was approved by the Biobank as was each investigator including all of the authors. UK Biobank supports extensive human subjects protection including written informed consent from each participant. The signed Materials Transfer Agreement between University of Illinois and UK Biobank specifies that we will abide by all their ethical standards.

### Study participants

UK Biobank is a prospective study with over 500,000 participants aged 40–69 years [8]. These participants were recruited during 2006–2010 from 22 assessment centers throughout the UK. The study is longitudinally collecting participants’ information, including data from questionnaires (self reports), physical measures (laboratory tests), and accelerometers (sensor records). It is representative of the national population for demographic and geographic considerations, although the entire cohort shows less disease and more education than the UK population at large [17]. Within the entire cohort, traditional risk factor associations agree for mortality outcomes with nationally representative cohort studies [31]. Thus the cohort dataset for sensor analysis is uniquely suitable for predictive models. UK Biobank provides accurate datasets for sensor input with physical activity and status output with health outcomes [19].

Our study focuses on the subset of 103,683 participants who agreed to wear a wrist-worn triaxial accelerometer, an Axivity AX3 sampling at 100Hz, continuously for 1 week [9]. These participants were aged 45–79 when data was collected in 2013–2015. We implemented inclusion/exclusion criteria shown in Fig 2. We exclude 257 participants for insufficient device wear time. Our analysis focused on walking intensity, so participants must have sufficient length of steady walking, as defined below. The Biobank software [19] divides sensor data into non-overlapping 30-second windows with activity labels. These are highly accurate, due to careful derivation from training set of representative participants who wore head-mounted cameras to visually identify activities. We included any participant with at least one session of steady walking, defined by 12 consecutive walking windows. Only windows labelled as walking were considered input data for feature extraction. We exclude 2758 participants for insufficient walking, which with other minor exclusions, yields total 100,655 participants for our analysis.

There were 2048 included deaths from UK Biobank field 40023, derived from the National Death Registry, which is a comprehensive curated dataset. We analyzed all-cause 5-year mortality, with sensor records from Jun 2013 to Dec 2015 and deaths until Dec 2019 to avoid COVID-19. For highest accuracy, our analysis used all qualifying participants, after trying different subsets including different age ranges. Most participants had known 5-year mortality.

### Steady walking in daily living

Walk tests are widely used to clinically evaluate status of cardiopulmonary patients. A standard assessment is the Six Minute Walk Test (6MWT), where a patient walks back and forth in a corridor for six minutes and their walked distance indicates their health status [6]. With COPD patients, this period is long enough so patients slow down in correlation with their status determined by spirometry [32]. Such walk tests are also used for CHF patients [33], who also exhibit Shortness of Breath on Exertion (SOBOE) [34]. We have previously shown with such cardiopulmonary patients that accelerometer sensors can measure slowdown/speedup with clinical accuracy, for predictive models of 6MWT distance and pulmonary function [27,16]. These were clinical experiments with COPD/CHF patients who performed 6MWT in hospital rehabilitation, with carried smartphones recording accelerometer sensors.

**Fig 6:**
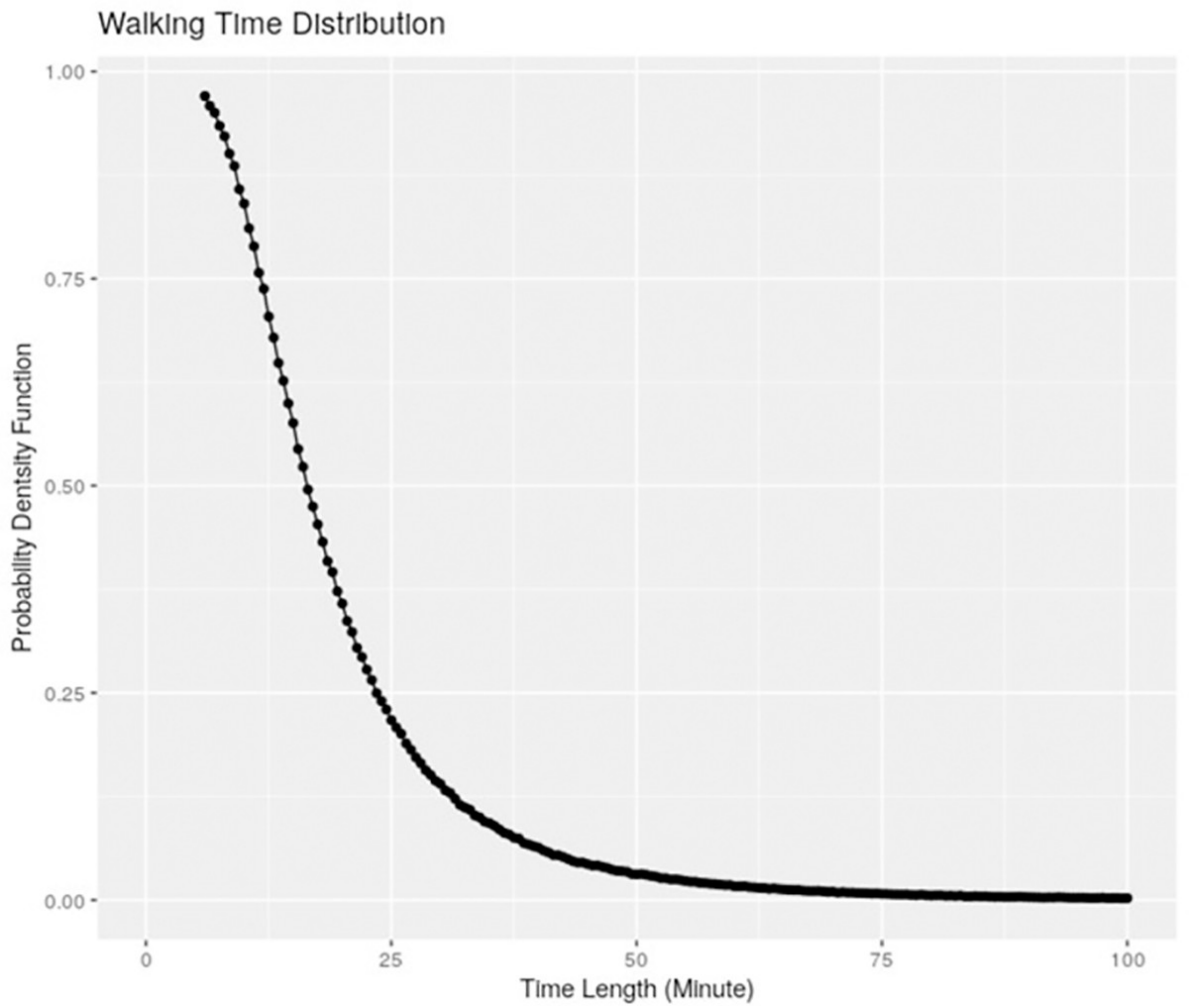
Walking Distribution of Labelled Sessions during Daily Living. Steady walking sessions only for included participants.

There is no current standard for walk tests during daily living. We chose 6 minutes as empirical lower bound for cardiopulmonary slowdown during *steady walking*, with relaxed criteria to allow longer periods. During daily living, a person may walk more slowly than when they are pushing hard during walk test, so it might take longer for them to experience SOBOE. Thus we require 12 consecutive walking windows to be the “equivalent of 6MWT”, and include all such labelled windows for included participants with at least 1 such session. For example, 4 consecutive walking windows would be excluded, while 12 consecutive windows or even 20 would be included. All included participants had at least 1 session of 6 minutes continuous walking during the 1 week, although only 10% walk half an hour in 6 minute sessions as shown in Fig 6.

### Mortality predictors: traditional categorical and accelerometer continuous

UK Biobank collected questionnaires during 2006–2010, when participants registered. We extract categorical features based upon self-reported answers and laboratory tests. We select 20 questions to characterize health status, including 7 Advanced Disease Conditions (ADC), 10 Modifiable Risk Factors (MRF), 3 Demographics. These are grouped into categories in Table 1. The original analysis for mortality risk using only categorical features found 13 features to be most discriminating [23], a subset of ours. S1 Table gives the deriving Biobank fields.

Accelerometer data is collected with the Axivity AX3 wrist-worn accelerometer, which collects 100Hz triaxial signals [9]. We follow the provided Biobank methods to extract features from raw data [19]. Our previous work showed raw sensor data was needed to predict status of pulmonary function in clinical studies with cardiopulmonary patients [16]. Model input was 6MWT sensor records, so more data points are needed than simply average sensor data over a labelled walking window.

Every 30 second time window is an epoch for measuring physical activity with single label, yielding 3000 3D acceleration points. The total number of possible epochs over 7 days is 20160. In each epoch, we extract a 76-dimensional feature vector, where each feature describes certain characteristics of motion patterns. Signal features are derived from time domain and frequency domain [35,36]. We use only features from time domain, since measuring walking intensity over time periods. We used 38 features from the Biobank software time domain [19], and computed another 38 features from their frequency domain following our previous work [16]. The 76 sensor features are listed in Table 2, while S2 Table gives their deriving formulas.

We added dimensional data, such as x-y-z, plus computing our own features from the frequency domain of Biobank software [19]. Our new features include those useful in other studies. These included RMS (root mean square) from our prior work [16], which is computable from FFT. Total activity count (TAC) is the overall feature provided by commercial fitness devices such as the research standard Actigraph GTX-3. We computed comparisons of most active periods to least active, such as MMCR (Maximum and Minimum average Cross Rate) and MCR (Mean Cross Rate), similar to physical activity profiles from activity duration methods. Full formulas for sensor features are given in S2 Table. The major features are defined in terms of ENMO, mean acceleration correlated with activity intensity such as walking versus standing. These include MPD and MAD, Mean Power Deviation and Mean Amplitude Deviation of accelerometer magnitude signals [37].

### Sensor data processing

The raw data was collected into 30-second windows over the entire week of recording, each window contained 3000 3-axis motion samples from field 90001. This comprised 25 terabytes. We analyzed this dataset using the Biocluster2 at the Carl R. Woese Institute for Genomic Biology, which has 72 nodes of Xeon Gold 6150s with 2 cores each of 2.7 GHz and 4GB memory. We computed 1037 batches of instances, where each batch consists of 100 instances. These instances covered the participant sensor record for each of almost 103,700 participants.

The total processing time for feature extraction was 3100 hours of compute time. It takes about 3 hours for each node to process each batch. The typical sensor processing used 50 nodes on the shared cluster, so the total real time was about 62 hours, or about 3 days. The steady state of extracted features is 1.2 TB, which we kept on the cluster storage as input to run the models.

### Mortality prediction models and survival function estimation

The prediction response is the time interval between end time of participant mortality and device wearing for sensor record. Since the outcome is time-to-event and subject to censoring, we utilize survival analysis [38]. Hence we consider the Cox proportional hazard model [39] and its penalized version [40]. We use the elastic net penalty [41], which consists of both l1 and l2 penalties of the coefficients, controlled by two tuning parameters α and λ. The method is implemented using the R package glmnet [42]. To select the optimal tuning, we consider α = 0, 0.5, 1 and use a grid of λvalues automatically selected by the glmnet package.

**Fig 7:**
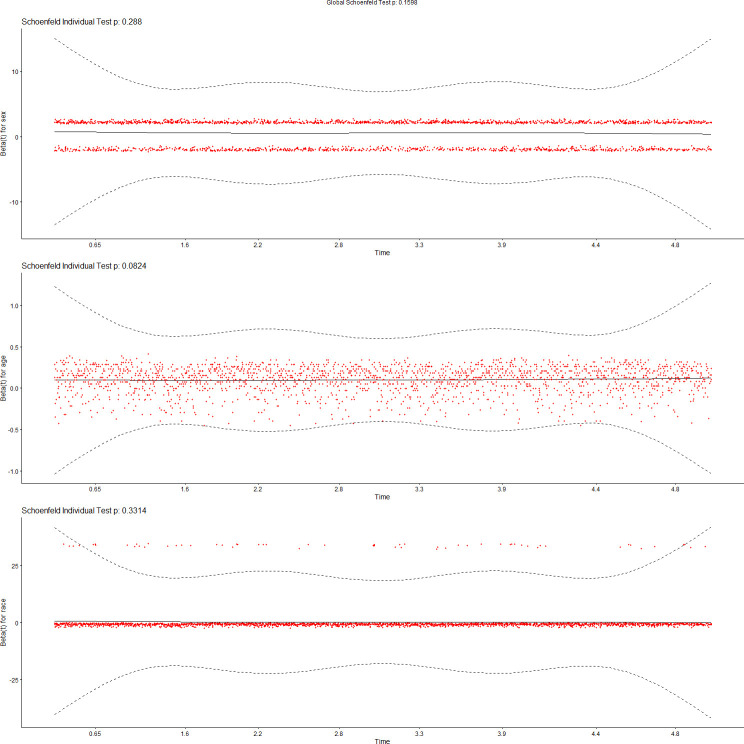
Feature tests for proportionality of risks: Demographics.

**Fig 8:**
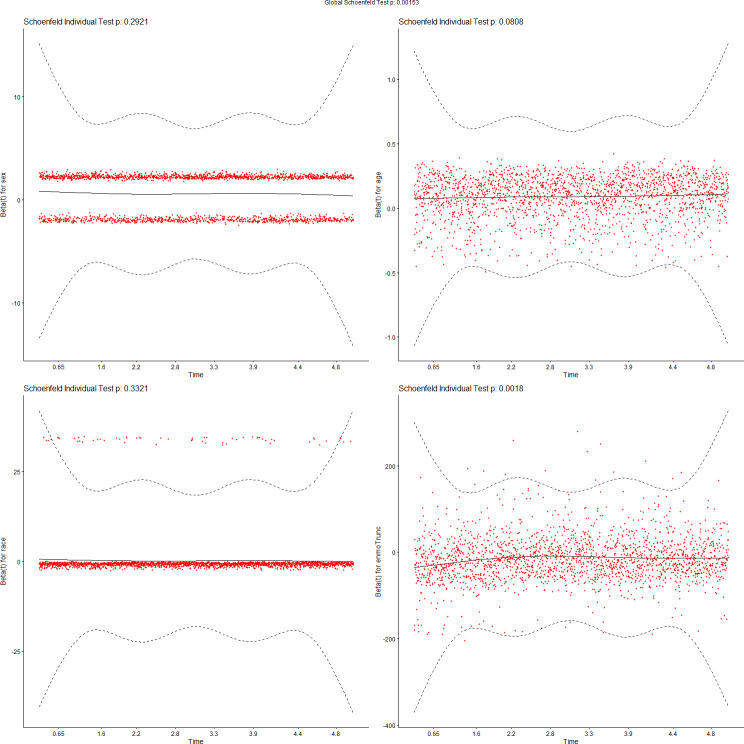
Feature tests for proportionality of risks: Demographics + ENMOtrunc.

**Fig 9:**
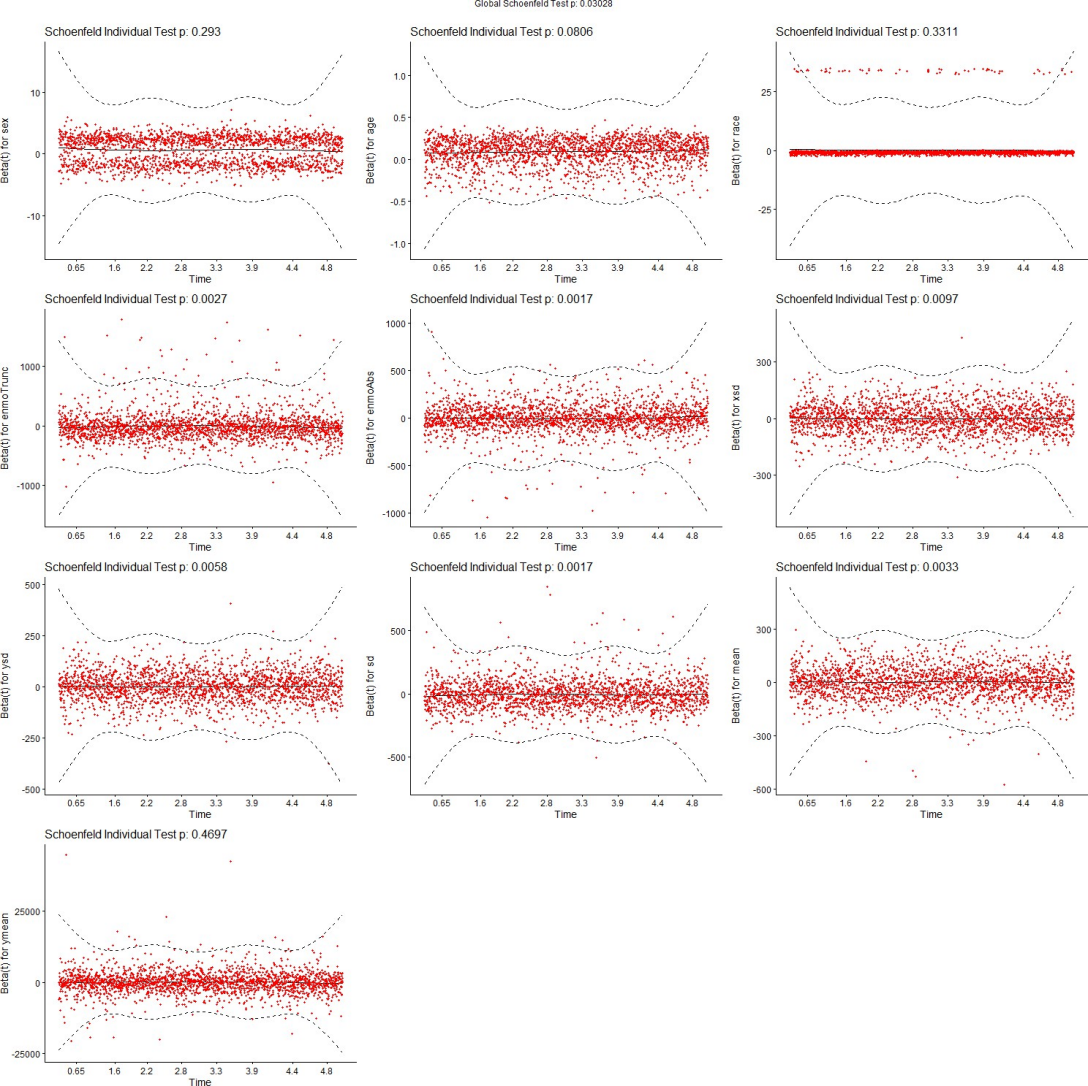
Feature tests for proportionality of risks: Min Model (10 top variables).

**Fig 10:**
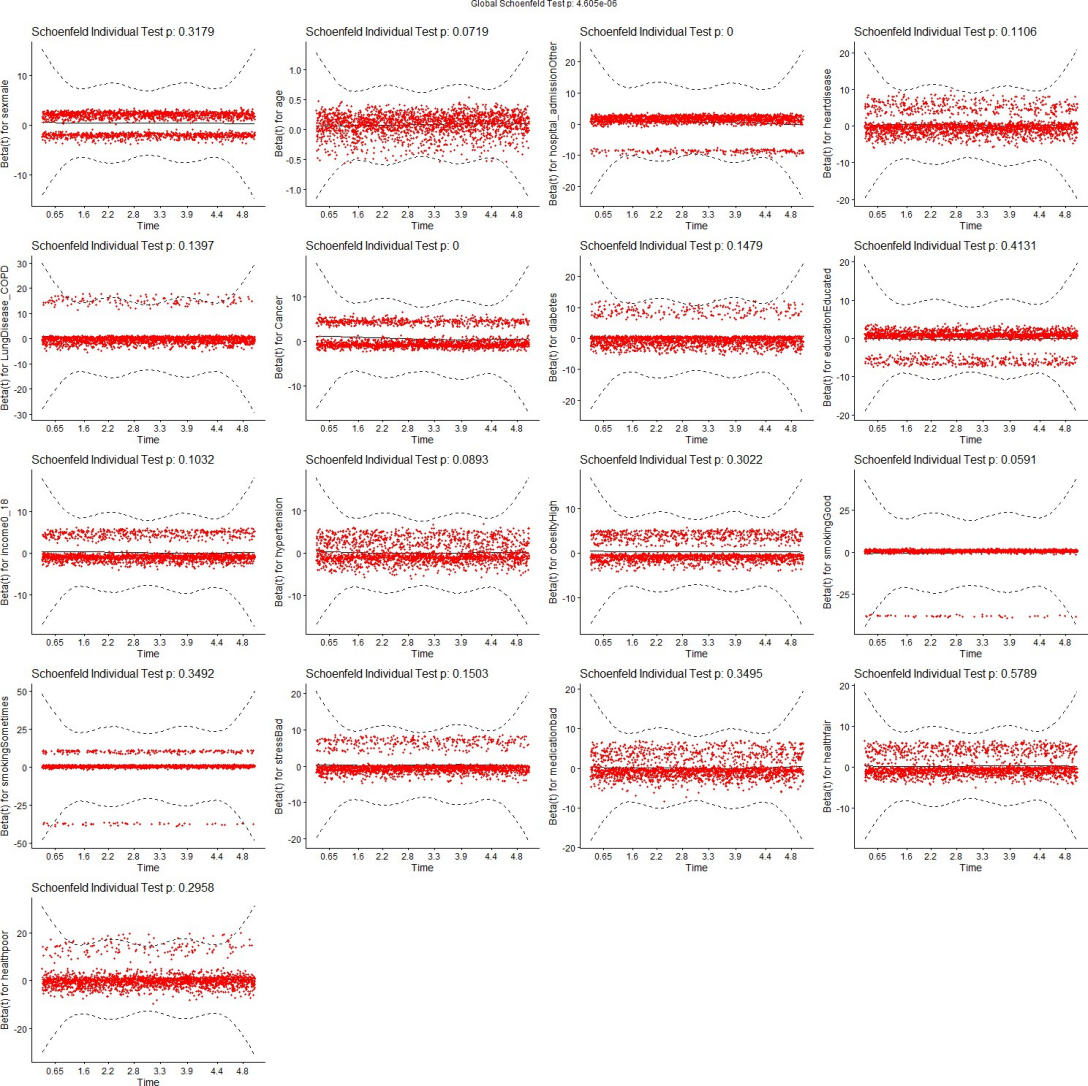
Feature tests for proportionality of risks: Max Model (select by lambda.1se).

We tested the assumptions of proportionality of risks in the Cox proportional hazard models using Schoenfeld residuals [43]. Figs 7–10 show the results of these tests. Fig 7 tests demographics features only, while Fig 8 adds ENMOtrunc. This represents our practical model with activity intensity. Fig 9 tests the Min Model, which includes the top 10 discriminating features from sensors and demographics, as listed in Table 4. Our practical model is a subset of these features. In all these figures, all variables pass the PH test (non-significant) if the significance threshold is at 0.001. Fig 10 tests our full featured Max Models with all categorical and all continuous features. In this figure, the p-values of 2 (categorical) variables, Hospital_Admissions (in Medical_Care) and Cancer (in Diagnosis), are close to 0 while all the other selected variables remain non-significant, thus passing the PH test. As Biobank field 40001 shows, Cancer is the largest cause of death in this cohort, producing almost exactly half of participant mortality. Note since our sample size is very large, significance level is probably not the most reliable determining factor, so visualization is likely more appropriate. By checking the residual plots, we can find visually that they center around the middle line very well. This indicates that the violation of the PH assumption is mild.

To test the accuracy of our results, we perform 10-fold cross-validation [44]. Cross-validation procedures are more stable than pre-fixing testing data since they enable all observed data to be used in evaluation steps. These are commonly used in data analytic procedures with machine learning methods [45]. The procedure 10-fold is the averaged result of performing 10 such pre-fixing procedures. In contrast, results derived from pre-fixing the testing data only once can be greatly affected by the randomness involved in choosing the test set.

We use a stratified 10-fold cross-validation approach, since the proportion of death is small (about 2% of participants). For each model with maximum follow-up length (1/2/3/4/5 years), we consider the data is randomly split into 10 equal-sized subsets. Each subset contains 1/10 of the live data (participants who are still alive or censored by the maximum follow-up time) and 1/10 of the dead data (participants who have died by the maximum follow-up time). With these 10 equal-size datasets, a single subset is utilized for testing the model and the remaining 9 subsets are used as training data. The cross-validation process is repeated 10 times with every subset used exactly once as the testing data. Finally, the 10 results from the folds can be averaged to produce a single estimation for a specific model with exact $alpha$ and $lambda$. For each $alpha$, the $lambda$ with best performance is selected from grid as model parameter.

In addition to the regularized Cox proportional hazards model, we fit other models to compare their performance. We adopt stepwise selection to choose variables. With fixed variables as input, we set the prediction performance as inclusion criteria to do stepwise forwards selecting over these variables. In every step, the variable that increases the C-index the most based on the previous selected variables is included in the group. The selection runs until the increment is less than a specific threshold. We have tested over traditional predictors and accelerometer derived predictors with threshold 0.01 and 0.001, to enable model evaluation.

The Concordance Index (C-index) is used to evaluate the model performance [46]. The C-index can be interpreted as the fraction of all pairs of subjects whose predicted survival times are correctly ordered as the observed survivals, while correcting for censoring. Hence, it is more sensible than other common criteria such as the overall accuracy or the Area under the Curve.

## Supporting information

S1 Table Categorical Encoding of features for cohort of 100,655 participants. Binary values are 1 for Bad (Health) and 0 for Good (Health). Range values have more choices, specific to each such field. For Date Fields, Censor Field if Date Report is AFTER Date Sensor, but Include Participant.
(DOCX)

S2 Table Continuous Encoding of formulas for accelerometer sensor features. (DOCX)

S3 Table Marginal Performance for all features ranked by C-index. (DOCX)

S1 Fig Lasso Model: Hierarchy tree average with red selected features. (DOCX)

S2 Fig Geographic Models: Site by site C-index computations. (DOCX)

## References

[1] Wang Y, Nie J, Ferrari G, Rey-Lopez JP, Rezende LFM. Association of Physical Activity Intensity with Mortality: A National Cohort Study of 403,681 US Adults. JAMA Intern Med 2021 (Feb);181(2):203–211. doi: 10.1001/jamainternmed.2020.6331 33226432 PMC7684516

[2] Ekelund U, Tarp J, Fagerland MW, Johannessen JS, Hansen BH, Jefferis BJ, et al. Joint associations of accelerometer measured physical activity and sedentary time with all-cause mortality: a harmonised meta-analysis in more than 44,000 middle-aged and older individuals. Br J Sports Med 2020 (Dec);54(24):1499–1506. doi: 10.1136/bjsports-2020-103270 33239356 PMC7719907

[3] Tabacu L, Ledbetter M, Leroux A, Crainiceanu C, Smirnova E. Quantifying the Varying Predictive Value of Physical Activity Measures Obtained from Wearable Accelerometers on All-Cause Mortality over Short to Medium Time Horizons in NHANES 2003–2006. Sensors (Basel) 2020 (Dec);21(1):4 (19pp). doi: 10.3390/s21010004 33374911 PMC7792606

[4] Studenski S, Perera S, Patel K, Rosano C, Faulkner K, Inzitari M, et al. Gait speed and survival in older adults. JAMA 2011 (Jan);305(1):50–58. doi: 10.1001/jama.2010.1923 21205966 PMC3080184

[5] Argyridou S, Zaccardi F, Davies M, Khunti K, Yates T. Walking pace improves all-cause and cardiovascular mortality risk prediction: A UK Biobank prognostic study. Eur J Prev Cardiol 2020 (Jul);27(10):1036–1044. doi: 10.1177/2047487319887281 31698963

[6] ATS Committee on Proficiency Standards for Clinical Pulmonary Function Laboratories. ATS statement: guidelines for the six-minute walk test. Am J Respir Crit Care Med 2002 (Jul);166(1):111–117. doi: 10.1164/ajrccm.166.1.at1102 12091180

[7] Grundtvig M, Eriksen-Volnes T, Ørn S, Slind EK, Gullestad L. 6 min walk test is a strong independent predictor of death in outpatients with heart failure. ESC (Eur Soc Cardio) Heart Fail 2020 (Oct);7(5):2904–2911. doi: 10.1002/ehf2.12900 32677748 PMC7524091

[8] Sudlow C, Gallacher J, Allen N, Beral V, Burton P, Danesh J, et al. UK Biobank: an open access resource for identifying the causes of a wide range of complex diseases of middle and old age. PLoS Med 2015 (Mar);12(3):e1001779 (10pp), doi: 10.1371/journal.pmed.1001779 . 25826379 PMC4380465

[9] Doherty A, Jackson D, Hammerla N, Plotz T, Olivier P, Granat MH, et al. Large Scale Population Assessment of Physical Activity Using Wrist Worn Accelerometers: The UK Biobank Study. PLoS One 2017 (Feb);12(2):e0169649 (14pp), doi: 10.1371/journal.pone.0169649 . 28146576 PMC5287488

[10] Hua A, Quicksall Z, Di C, Motl R, LaCroix AZ, Schatz BR, Buchner DM. Accelerometer-based predictive models of fall risk in older women: a pilot study. Nature (NPJ) Digital Med 2018 (Jul);1:25 (8pp). doi: 10.1038/s41746-018-0033-5 31304307 PMC6550179

[11] Schatz BR. Population measurement for health systems. Nature (NPJ) Digital Med 2018 (Jan); 1:4 (4pp). 10.1038/s41746-017-0004-2. 31304348 PMC6550166

[12] Pew Research Center. Demographics of Mobile Devices: Mobile Fact Sheet, 2021 (Apr 7). Available from: https://www.pewresearch.org/internet/fact-sheet/mobile/.

[13] Pew Research Center. About one-in-five Americans use a smart watch or fitness tracker. 2020 (Jan 9). Available from: https://www.pewresearch.org/fact-tank/2020/01/09/about-one-in-five-americans-use-a-smart-watch-or-fitness-tracker/.

[14] O’Dea S. Smartphone ownership in the United Kingdom (UK) 2012–2021. 2021 (May 21). Available from: https://www.statista.com/statistics/271851/smartphone-owners-in-the-united-kingdom-uk-by-age/.

[15] O’Dea S. Global smartphone penetration rate as share of population from 2016 to 2020. 2021 (Dec 16). Available from: https://www.statista.com/statistics/203734/global-smartphone-penetration-per-capita-since-2005/.

[16] Cheng Q, Juen J, Bellam S, Fulara N, Close D, Silverstein JC, Schatz B. Predicting Pulmonary Function from Phone Sensors. Telemed J E Health. 2017 (Nov);23(11):913–919. doi: 10.1089/tmj.2017.0008 . https://www.liebertpub.com/doi/full/10.1089/tmj.2017.0008. 28300524 PMC5684658

[17] Fry A, Littlejohns TJ, Sudlow C, Doherty N, Adamska L, Sprosen T, et al. Comparison of Sociodemographic and Health-Related Characteristics of UK Biobank Participants with Those of the General Population. Am J Epidemiol 2017 (Nov);186(9):1026–1034. doi: 10.1093/aje/kwx246 . 28641372 PMC5860371

[18] Juen J, Cheng Q, Schatz B. A natural walking monitor for pulmonary patients using mobile phones. IEEE J Biomed Health Inform 2015 (Jul);19(4):1399–405. doi: 10.1109/JBHI.2015.2427511 . 25935052

[19] Willetts M, Hollowell S, Aslett L, Holmes C, Doherty A. Statistical machine learning of sleep and physical activity phenotypes from sensor data in 96,220 UK Biobank participants. Sci Rep 2018 (May);8(1):7961 (10pp). doi: 10.1038/s41598-018-26174-1 . 29784928 PMC5962537

[20] van Hees VT, Gorzelniak L, Dean León EC, Eder M, Pias M, Taherian S, et al. Separating movement and gravity components in an acceleration signal and implications for the assessment of human daily physical activity. PLoS One 2013 (Apr);8(4):e61691 (10pp). doi: 10.1371/journal.pone.0061691 . 23626718 PMC3634007

[21] Tibshirani R. Regression shrinkage and selection via the lasso. J Royal Statistical Soc: Series B Methodological 1996;58(1):267–288.

[22] Bakrania K, Yates T, Rowlands AV, Esliger DW, Bunnewell S, Sanders J, et al. Intensity Thresholds on Raw Acceleration Data: Euclidean Norm Minus One (ENMO) and Mean Amplitude Deviation (MAD) Approaches. PLoS One 2016 (Oct);11(10):e0164045 (16pp). doi: 10.1371/journal.pone.0164045 . 27706241 PMC5051724

[23] Ganna A, Ingelsson E. 5 year mortality predictors in 498,103 UK Biobank participants: a prospective population-based study. Lancet 2015 (Aug);386(9993):533–540. doi: 10.1016/S0140-6736(15)60175-1 . 26049253

[24] Wang R, Bishwajit G, Zhou Y, Wu X, Feng D, Tang S, et al. Intensity, frequency, duration, and volume of physical activity and its association with risk of depression in middle- and older-aged Chinese: Evidence from the China Health and Retirement Longitudinal Study, 2015. PLoS One 2019 (Aug);14(8):e0221430 (16pp). doi: 10.1371/journal.pone.0221430 . 31425559 PMC6699736

[25] Laursen AH, Kristiansen OP, Marott JL, Schnohr P, Prescott E. Intensity versus duration of physical activity: implications for the metabolic syndrome. A prospective cohort study. BMJ Open 2012;2:e001711. doi: 10.1136/bmjopen-2012-001711 . 23045359 PMC3488727

[26] Leroux A, Xu S, Kundu P, Muschelli J, Smirnova E, Chatterjee N, et al. Quantifying the Predictive Performance of Objectively Measured Physical Activity on Mortality in the UK Biobank. J Gerontol: A Biol Sci Med Sci 2021 (Jul) 13; 76(8):1486–1494. doi: 10.1093/gerona/glaa250 . 33000171 PMC8277083

[27] Juen J, Cheng Q, Prieto-Centurion V, Krishnan JA, Schatz B. Health monitors for chronic disease by gait analysis with mobile phones. Telemed J E Health. 2014 (Nov);20(11):1035–1041. doi: 10.1089/tmj.2014.0025 . https://www.liebertpub.com/doi/10.1089/tmj.2014.0025. 24694291 PMC4229704

[28] Cheng Q, Juen J, Hsu-Lumetta J, Schatz B. Predicting Transitions in Oxygen Saturation Using Phone Sensors. Telemed J E Health 2016 (Feb);22(2):132–137. doi: 10.1089/tmj.2015.0040 . https://www.liebertpub.com/doi/10.1089/tmj.2015.0040. 30175953 PMC4744879

[29] Lear S, Hu W, Rangarajan S, Gasivic D, Leong D, Iqbal R, et al. The effect of physical activity on mortality and cardiovascular disease in 130,000 people from 17 high-income, middle-income, and low-income countries: the PURE study. Lancet 390: 2643–2653 (Dec 16, 2017). doi: 10.1016/S0140-6736(17)31634-3 . 28943267

[30] Schatz BR, Berlin RB. Healthcare Infrastructure: Health Systems for Individuals and Populations. London: Springer-Verlag Series in Health Informatics; 2011.

[31] Batty GD, Gale CR, Kivimäki M, Deary IJ, Bell S. Comparison of risk factor associations in UK Biobank against representative, general population based studies with conventional response rates: prospective cohort study and individual participant meta-analysis. BMJ 2020 (Feb); 368:m131. doi: 10.1136/bmj.m131 . 32051121 PMC7190071

[32] Lahousse L, Verlinden VJ, van der Geest JN, Joos GF, Hofman A, Stricker BH, et al. Gait patterns in COPD: the Rotterdam Study. Eur Respir J 2015 (Jul);46(1):88–95. doi: 10.1183/09031936.00213214 . 25700390

[33] Jehn M, Schmidt-Trucksaess A, Schuster T, Hanssen H, Halle M, Kohler F. Accelerometer-based quantification of 6-minute walk test performance in patients with chronic heart failure: Applicability in telemedicine. J Card Fail 2009;15:334–340. doi: 10.1016/j.cardfail.2008.11.011 19398082

[34] Clark AL. Origin of symptoms in chronic heart failure. Heart. 2006 (Jan);92(1):12–6. doi: 10.1136/hrt.2005.066886 16159969 PMC1860978

[35] Zhang S, Rowlands AV, Murray P, Hurst TL. Physical activity classification using the GENEA wrist-worn accelerometer. Med Sci Sports Exerc 2012 (Apr);44(4):742–748. doi: 10.1249/MSS.0b013e31823bf95c 21988935

[36] Ellis K, Kerr J, Godbole S, Staudenmayer J, Lanckriet G. Hip and Wrist Accelerometer Algorithms for Free-Living Behavior Classification. Med Sci Sports Exerc 2016 (May);48(5):933–940. doi: 10.1249/MSS.0000000000000840 26673126 PMC4833514

[37] Marin F, Lepetit K, Fradet L, Hansen C, Ben Mansour K. Using accelerations of single inertial measurement units to determine the intensity level of light-moderate-vigorous physical activities: Technical and mathematical considerations. J Biomech 2020 (Jun);107:109834. doi: 10.1016/j.jbiomech.2020.109834 Epub 2020 May 12. 32517856

[38] Fleming TR, Harrington DP. Counting Processes and Survival Analysis. 2^nd^ ed. New York: Wiley-Interscience; 2013.

[39] Cox DR. Regression models and life tables (with discussion). J Royal Statistical Soc 1972;34(2):187–220.

[40] Simon N, Friedman J, Hastie T, Tibshirani R. Regularization paths for Cox’s proportional hazards model via coordinate descent. J Statistical Software 2011 (Mar);39(5):1. doi: 10.18637/jss.v039.i05 27065756 PMC4824408

[41] Zou H, Hastie T. Regularization and variable selection via the elastic net. J Royal Statistical Soc: Series B Methodological 2005 (Apr);67(2):301–320.

[42] Friedman J, Hastie T, Tibshirani R. Regularization paths for generalized linear models via coordinate descent. J Statistical Software 2010;33(1):1. 20808728 PMC2929880

[43] Grambsch PM, Therneau TM. Proportional hazards tests and diagnostics based on weighted residuals. Biometrika 1994; 81(3), 515–526.

[44] Kohavi R. A study of cross-validation and bootstrap for accuracy estimation and model selection. In Ijcai 1995 (Aug 20);14(2):1137–1145.

[45] Hastie T, Tibshirani R, Friedman J. The Elements of Statistical Learning: Data Mining, Inference, and Prediction. 2^nd^ ed. New York: Springer Series in Statistics; 2016.

[46] Harrell FE, Califf RM, Pryor DB, Lee KL, Rosati RA. Evaluating the yield of medical tests. JAMA 1982 (May) 14;247(18):2543–2546. 7069920

