# Supplementary material for: Population analysis of mortality risk: Predictive models from passive monitors using motion sensors for 100,000 UK Biobank participants
Source: PLOS Digit Health. 2022 Oct 20;1(10):e0000045. doi: 10.1371/journal.pdig.0000045 (PMC9931283; doi:10.1371/journal.pdig.0000045)
Supplement: S1 Table — Binary values are 1 for Bad (Health) and 0 for Good (Health). Range values have more choices, specific to each such field. For Date Fields, Censor Field if Date Report is AFTER Date Sensor, but Include Participant. (DOCX) [file pdig.0000045.s001.docx]

**S1 Table**. **Categorical Encoding of features for cohort of 100,655 participants.**

Binary values are 1 for Bad (Health) and 0 for Good (Health).

Range values have more choices, specific to each such field.

For Date Fields, Censor Field if Date Report is AFTER Date Sensor, but Include Participant.

| **Feature_Name** | | **Biobank_Field** | **Summary_Statistics** | |
| --- | --- | --- | --- | --- |
| **Demographics** | |  |  | |
| Age | | 34 | DateSensorWear 90010 – YearOfBirth 34 | |
| Sex | | 31 | (Gender) male : 43981 / female : 56674 | |
| Race | | 21000 | (Ethnicity) others : 3493 / white : 97162 | |
| **Diagnosis** | |  |  | |
| Cardiovascular Disease | | 8 fields | 1 : 7083 / 0 : 93572 (merge w/o duplicates) | |
| Myocardial Infarction | | 131298 | 1 : 2164 / 0 : 98491 (Date Report) | |
| Angina Pectoris | | 131296 | 1 : 3845 / 0 : 96810 (Date Report) | |
| Congestive Heart Failure | | 131354 | 1 : 740 / 0 : 99915 (Date Report) | |
| Ischemic Heart Disease | | 131306 | 1 : 4248 / 0 : 96407 (Date Report) | |
| Stroke, Cerebral Infarction | | 131366 | 1 : 441 / 0 : 100214 (Date Report) | |
| Stroke, not Infarction | | 131368 | 1 : 1021 / 0 : 99634 (Date Report) | |
| Stenosis, Precerebral Arteries | | 131370 | 1 : 130 / 0 : 100525 (Date Report) | |
| Stenosis, Cerebral Arteries | | 131372 | 1 : 13 / 0 : 100642 (Date Report) | |
| Pulmonary Disease (COPD) | | 42016 | 1 : 1685 / 0 : 98970 (Date Report) | |
| Cancer | | 40005 | 1 : 14301 / 0 : 86354 (Date Report) | |
| Cancer Behavior (Malignant) | | 40012 | 1 : 12825 / 0 : 87830 (Date Report) | |
| Diabetes | | 3 fields | 1 : 4606 / 0 : 96049 (merge w/o duplicates) | |
| Diabetes Type1 | | 130706 | 1 : 446 / 0 : 100209 (Date Report) | |
| Diabetes Type2 | | 130708 | 1 : 3389 / 0 : 97266 (Date Report) | |
| Diabetes type unspecified | | 130714-0.0 | 1 : 3237 / 0 : 97418 (Date Report) | |
| **Medicalcare** |  |  | |  |
| Operation | 2 fields | NAValue : 48 / Unknown : 656 / Operation : 65212 / NoOperation : 34739 | |  |
| Operation_male | 2415 | NAValue : 56697 / Unknown : 254 / Operation : 27810 / NoOperation : 15894 | |  |
| Operation_female | 2844 | NAValue : 44006 / Unknown : 402 / Operation : 37402 / NoOperation : 18845 | |  |
| Hospital Admission | 41249 | NAValue : 20471 / Emergency : 5815 / Other : 74369 | |  |
| Falls in last year | 2296 | NAValue : 45 / Unknown : 107 / High risk : 5286 / Low risk : 95217 | |  |
| **Screening** |  |  | |  |
| Hypertension | 131286 | 1 : 15946 / 0 : 84709 (Date Report) | |  |
| Cholesterol | 30690 | NAValue : 5892 / High : 39787 / Normal : 54976 | |  |
| Obesity | 21001 | NAValue : 226 / High : 19598 / Normal : 80831 | |  |
| Medications | 137 | NAValue : 26 / Bad : 14642 / Good : 19156 / Problem : 66831 | |  |
| **Habit** |  |  | |  |
| Alcohol | 1558 | NAValue : 45 / Unknown : 38 / Daily : 23025 / Week34 : 26145 / Week12 : 25228 / Month13 : 10944 / Special : 9543 / Never : 5687 | |  |
| Smoking | 1239 | NAValue : 44 / Unknown : 21 / Bad : 2239/ Sometimes : 4760 / Good : 93591 | |  |
| Stress | 6145 | NAValue : 595 / Unknown : 263 / Bad : 8287 / Serious Other : 34021/ Good : 57489 | |  |
| **Lifestyle** |  |  | |  |
| Health | 2178 | NAValue : 45 / Unknown : 198 / excellent : 21807 / good : 60153 / fair : 15881 / poor : 2571 | |  |
| Education | 6138 | NAValue : 594 / Unknown : 8716 / Educated : 91345 | |  |
| Income | 738 | NAValue : 704 / Unknown : 9704 / 0_18 : 13284 / 18_31 : 21798 / 31_52 : 25894 / 52_100 : 22672 / 100 : 6599 | |  |
