# Supplementary material for: Population analysis of mortality risk: Predictive models from passive monitors using motion sensors for 100,000 UK Biobank participants
Source: PLOS Digit Health. 2022 Oct 20;1(10):e0000045. doi: 10.1371/journal.pdig.0000045 (PMC9931283; doi:10.1371/journal.pdig.0000045)
Supplement: S2 Table — (DOCX) [file pdig.0000045.s002.docx]

**S2 Table**. **Continuous Encoding of formulas for accelerometer sensor features.**

| Variable Name | Explanation | Formula |
| --- | --- | --- |
| ENMOtrunc | truncated Euclidean Norm Minus One | $max\left( 0,\sqrt{x_{i}^{2}+ y_{i}^{2}+ z_{i}^{2}}-1 \right)$ |
| ENMOabs | absolute Euclidean Norm Minus One | $\left\vert\sqrt{x_{i}^{2}+ y_{i}^{2}+ z_{i}^{2}}-1 \right\vert$ |
| Mean  (xMean, yMean, zMean) | Mean of vector magnitude  (x / y / z axis) | $\left( \sum_{i=1}^{n} {enmoTrunc}_{i} \right)/n$  (  $\left( \sum_{i=1}^{n} x_{i} (y_{i} or z_{i} \right)/n$  ) |
| Sd  (xSd, ySd, zSd) | Standard deviation of vector magnitude (x / y / z axis) | $\sqrt{\left( \sum_{i=1}^{n} enmoTrunc_{i} - mean \right)^{2}/n}$ |
| RMS  (xRMS, yRMS, zRMS) | Root Mean Square | $\sqrt{\left( \sum_{i=1}^{n} {enmoTrunc}_{i}^{2} \right)/n}$  ($\sqrt{\left( \sum_{i=1}^{n} x_{i}^{2} ( y_{i}^{2} or z_{i}^{2} \right)/n}$  ) |
| TAC  (xTAC, yTAC, zTAC) | Total Activity | $\sum_{i=1}^{n} \left\vert{enmoTunc}_{i} \right\vert$  (  $\sum_{i=1}^{n} \left\vert x_{i}\left( y_{i}orz_{i} \right) \right\vert$  ） |
| MCR  (xMCR, yMCR, zMCR) | Mean Cross Rate | $(\sum_{i=1}^{n-1} \mathbb{1\{ (}enmoTrunc_{i} - mean )(enmoTrunc_{i} - mean) < 0\})/(n-1)$ |
| MMCR  (xMMCR, yMMCR, zMMCR) | Maximum and Minimum Average Cross Rate | $(\sum_{i=1}^{n-1} \mathbb{1\{ (}enmoTrunc_{i} - (max - min)/2 )(enmoTrunc_{i} - (max - min)/2) < 0\})/(n-1)$ |
| MAD | Mean Amplitude Deviation | $\left( \sum_{i=1}^{n} \vert enmoTrunc_{i}-mean\vert\right)/n$ |
| MPD | Mean Power Deviation | $\left( \sum_{i=1}^{n} {\vert enmoTrunc_{i}-mean\vert}^{1.5} \right)/n^{1.5}$ |
| Min  (xMin, yMin, zMin) | Minimum of vector | ${min}_{i=1,\ldots,n}{enmoTrunc}_{i}$  (  ${min}_{i=1,\ldots,n}x_{i}\left( y_{i}orz_{i} \right)$  ) |
| 25thp  (x25thp, y25thp, z25thp) | 0.25 percentile | 25% rank of ${enmoTrunc}_{i} (i =1,...,n)$  (  25% rank of $x_{i} ( y_{i} or z_{i} ) (i = 1,...,n)$  ) |
| Median  (xMedian, yMedian, zMedian) | median | 50% rank of ${enmoTrunc}_{i} (i =1,...,n)$  (  50% rank of $x_{i} ( y_{i} or z_{i} ) (i = 1,...,n)$  ) |
| 75thp  (x75thp, y75thp, z75thp) | 0.75 percentile | 75% rank of ${enmoTrunc}_{i} (i =1,...,n)$  (  75% rank of $x_{i} ( y_{i} or z_{i} ) (i = 1,...,n)$  ) |
| Max  (xMax, yMax, zMax) | maximum | ${max}_{i=1,\ldots,n}{enmoTrunc}_{i}$  (  ${max}_{i=1,\ldots,n}x_{i}\left( y_{i}orz_{i} \right)$  ) |
| xRange / yRange / zRange | Range of x / y / z axis | $\left( xmax-xmin \right)/\left( ymax-ymin \right)/\left( zmax-zmin \right)$ |
