# Supplementary material for: Population analysis of mortality risk: Predictive models from passive monitors using motion sensors for 100,000 UK Biobank participants
Source: PLOS Digit Health. 2022 Oct 20;1(10):e0000045. doi: 10.1371/journal.pdig.0000045 (PMC9931283; doi:10.1371/journal.pdig.0000045)
Supplement: S3 Table — (DOCX) [file pdig.0000045.s003.docx]

**S3 Table**. **Marginal Performance for all features ranked by C-index.**

| **Name** | **C-index** |
| --- | --- |
| Age | 0.699329 |
| ENMOtrunc | 0.639477 |
| MPD | 0.637168 |
| MAD | 0.630199 |
| ENMOabs | 0.629914 |
| Sd | 0.626054 |
| RMS | 0.615782 |
| 75thp | 0.612617 |
| yRange | 0.612016 |
| Mean | 0.611020 |
| TAC | 0.610951 |
| xRange | 0.610390 |
| Ymin | 0.609938 |
| Income | 0.608182 |
| Median | 0.606270 |
| Max | 0.604575 |
| Ymax | 0.603579 |
| zRange | 0.603381 |
| Xmax | 0.602969 |
| Xmin | 0.600941 |
| Zmin | 0.600338 |
| Medication | 0.598063 |
| 25thp | 0.597637 |
| xRMS | 0.597255 |
| Xsd | 0.597190 |
| Zmax | 0.595800 |
| xTAC | 0.590543 |
| Hypertension | 0.589080 |
| Health | 0.586785 |
| Sex | 0.584188 |
| x25thp | 0.583206 |
| x75thp | 0.581649 |
| Hospital_admission | 0.580935 |
| Cancer | 0.580095 |
| Ysd | 0.579393 |
| yRMS | 0.579341 |
| Zsd | 0.577168 |
| zRMS | 0.577154 |
| yTAC | 0.572009 |
| zTAC | 0.567890 |
| Heartdisease | 0.565598 |
| y75thp | 0.565253 |
| y25thp | 0.562617 |
| Stress | 0.559431 |
| z75thp | 0.556267 |
| z25thp | 0.555108 |
| Obesity | 0.547041 |
| min | 0.546833 |
| Alcohol | 0.545644 |
| Education | 0.542326 |
| Diabetes | 0.536643 |
| sdpitch | 0.533721 |
| sdyaw | 0.531501 |
| Smoking | 0.530064 |
| pitchg | 0.529827 |
| Operation | 0.526503 |
| LungDisease_COPD | 0.526217 |
| skew | 0.522987 |
| Cholesterol | 0.520420 |
| xautocorr | 0.516339 |
| Falls | 0.513014 |
| sdroll | 0.512742 |
| ymedian | 0.509274 |
| xyCov | 0.508795 |
| corrxy | 0.507807 |
| ymean | 0.507165 |
| avgyaw | 0.506790 |
| yzCov | 0.506411 |
| corryz | 0.505973 |
| xmean | 0.505578 |
| zmean | 0.505249 |
| avgpitch | 0.504397 |
| kurt | 0.503650 |
| yautocorr | 0.503615 |
| coefvariation | 0.503595 |
| xMCR | 0.503576 |
| Race | 0.50246 |
| zmedian | 0.502363 |
| MMCR | 0.498242 |
| zcoefvariation | 0.497630 |
| rollg | 0.496389 |
| xMMCR | 0.495558 |
| corrxz | 0.494586 |
| ycoefvariation | 0.494541 |
| xzCov | 0.492141 |
| avgroll | 0.491459 |
| autocorr | 0.490850 |
| xcoefvariation | 0.490604 |
| yawg | 0.486496 |
| yMCR | 0.484588 |
| xmedian | 0.484326 |
| zautocorr | 0.482311 |
| yMMCR | 0.478037 |
| zMMCR | 0.476288 |
| MCR | 0.475580 |
| zMCR | 0.469512 |
