# Supplementary figures and images for: Population analysis of mortality risk: Predictive models from passive monitors using motion sensors for 100,000 UK Biobank participants
Source: PLOS Digit Health. 2022 Oct 20;1(10):e0000045. doi: 10.1371/journal.pdig.0000045 (PMC9931283; doi:10.1371/journal.pdig.0000045)

**S1 Fig**. **Lasso Model: hierarchy tree average with red selected features.**


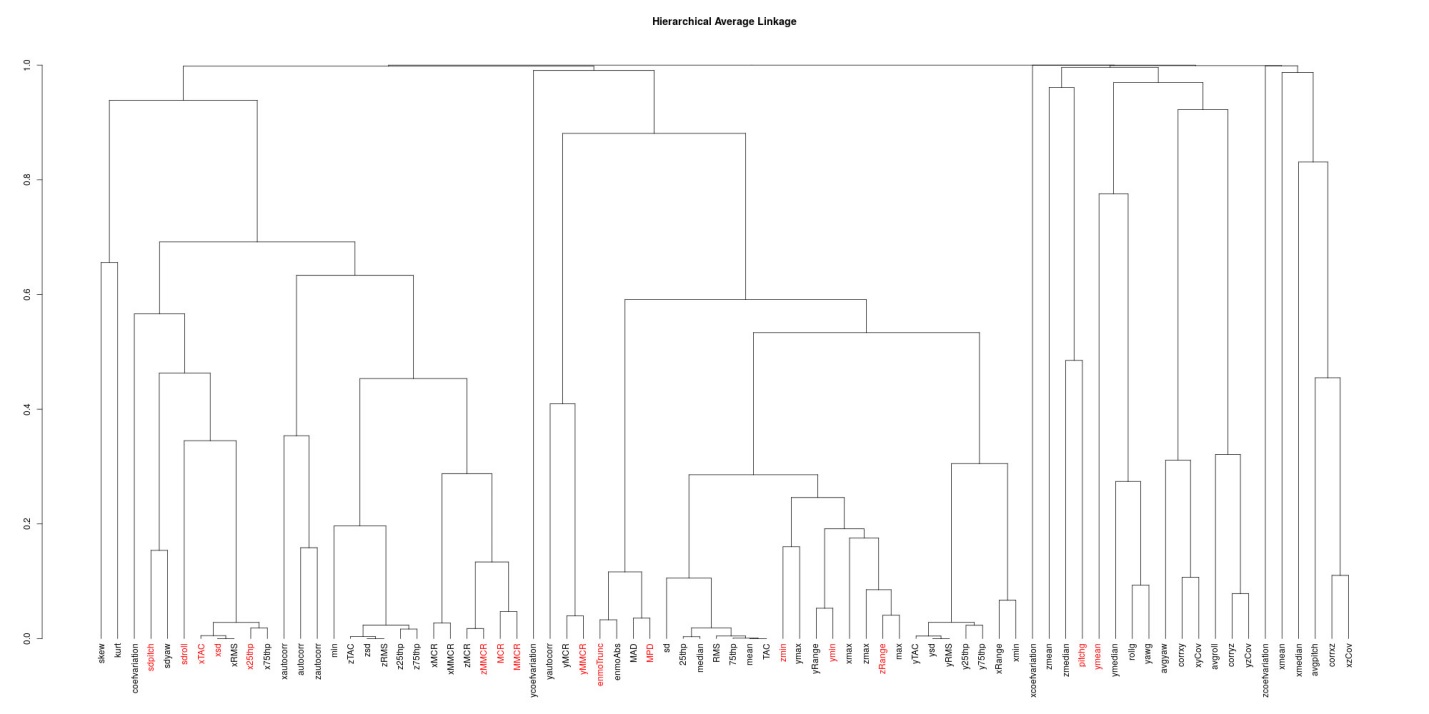

Supplement: S1 Fig — (DOCX) [file pdig.0000045.s004.docx]

**S2 Fig**. **Geographic Models: site by Site C-index computations.**


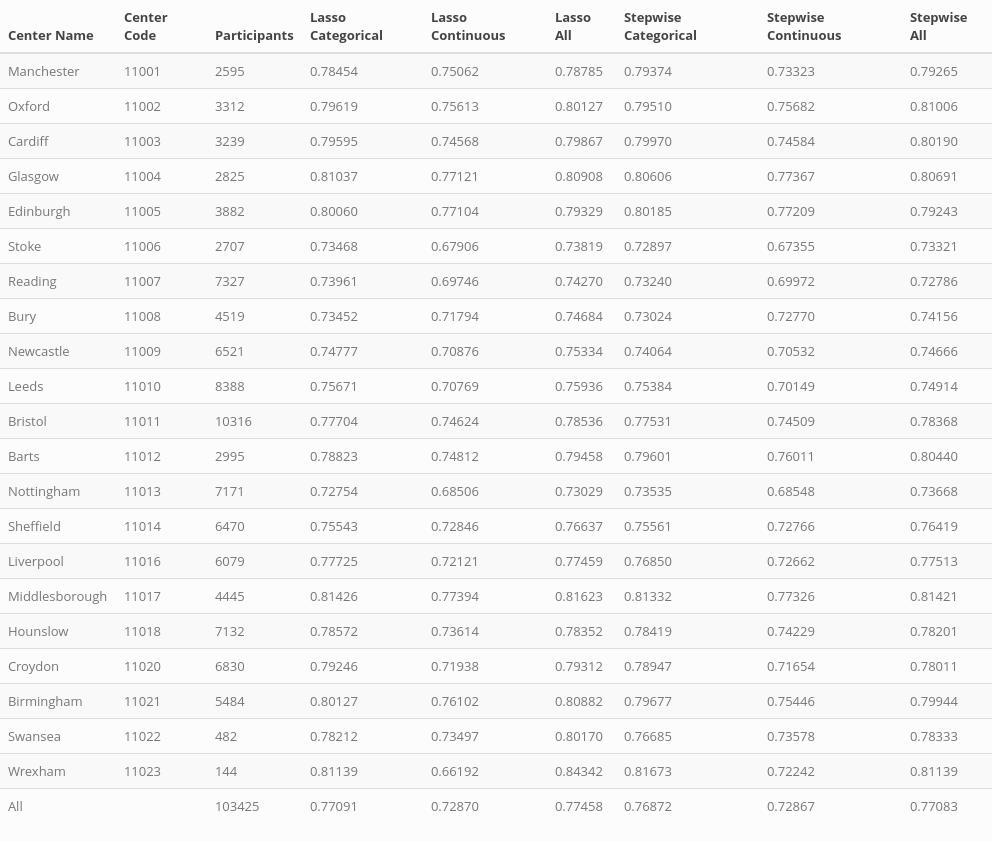

Supplement: S2 Fig — (DOCX) [file pdig.0000045.s005.docx]
